# A nationwide cross-sectional survey on prevalence, management and pharmacoepidemiology patterns on hypertension in Chinese patients with chronic kidney disease

**DOI:** 10.1038/srep38768

**Published:** 2016-12-20

**Authors:** Wen Zhang, Wei Shi, Zhangsuo Liu, Yong Gu, Qinkai Chen, Weijie Yuan, Yanlin Zhang, Li Gong, Rong Zhou, Mingxu Li, Hong Cheng, Jian Liu, Jun Cen, Chaoxing Huang, Yeping Ren, Peiju Mao, Changying Xing, Fuyuan Hong, Dongsheng Jiang, Li Wang, Gang Xu, Jianshe Liu, Nan Chen

**Affiliations:** 1 Department of Nephrology, Ruijin Hospital Shanghai Jiao Tong University School of Medicine, Shanghai, 200025, China; 2 Department of Nephrology, Guangdong General Hospital, Guangzhou, 510030, China; 3 Department of Nephrology, The First Affiliated Hospital of Zhengzhou University, Zhengzhou, 450052 China; 4 Department of Nephrology, The Fifth Affiliated Hospital of Fudan University, Shanghai, 200240, China; 5 Department of Nephrology, The First Affiliated Hospital of Nanchang University, Nanchang, 330006, China; 6 Department of Nephrology, The First Affiliated Hospital of Shanghai Jiao Tong University, Shanghai, 200080, China; 7 Department of Nephrology, The First Affiliated Hospital of Xiamen University, Xiamen, 361003, China; 8 Department of Nephrology, Department of Nephrology, Inner Mongolia People’s Hospital, Inner Mongolia, China; 9 Department of Nephrology, Shanghai Yangpu Hospital, Tongji University, Shanghai, 200090, China; 10 Department of Nephrology, PLA Navy General Hospital, Beijing, 100048, China; 11 Department of Nephrology, Beijing Anzhen Hospital Capital Medical University, Beijing, 100029, China; 12 Department of Nephrology, The First Affiliated Hospital of Xinjiang Medical University, Xinjiang, China; 13 Department of Nephrology, Shanghai Construction Group Hospital, Shanghai, 200083, China; 14 Department of Nephrology, The First Affiliated Hospital of Wenzhou Medical University, Wenzhou, 325000, China; 15 Department of Nephrology, The Second Affiliated Hospital of Harbin Medical University, Harbin, China; 16 Department of Nephrology, Tongren Hospital, Shanghai Jiao Tong University School of Medicine, Shanghai, 200336, China; 17 Department of Nephrology, Jiangsu Province Hospital, Nanjing, 210029, China; 18 Department of Nephrology, Fujian Provincial Hospital, Fuzhou, 350001, China; 19 Department of Nephrology, Jiangsu Taizhou People’s Hospital, Taizhou, 225300, China; 20 Department of Nephrology, Sichuan Provincial People’s Hospital, Sichuan, 610072, China; 21 Department of Nephrology, Tongji Hospital, Tongji Medical College, Huazhong University of Science and Technology, Wuhan, 430030, China; 22 Department of Nephrology, Union Hospital, Huazhong University of Science and Technology, Wuhan, 430022, China

## Abstract

Limited data are available on epidemiology and drug use in Chinese hypertensive patients with chronic kidney disease (CKD). We determined the prevalence; awareness, treatment, and control rates of hypertension; anti-hypertensive use, expenditure pattern; and factors associated with hypertension prevalence and control in Chinese patients with CKD. This was one of the largest cross-sectional surveys that enrolled 6079 CKD participants (mean age, 51.0 ± 16.37 years) with or without hypertension from 22 centres across China. The prevalence, awareness, and treatment rates were 71.2%, 95.4%, and 93.7%, respectively. Control rates 1 and 2 (Blood pressure, BP <140/90 and <130/80 mmHg) were 41.1% and 15.0%, respectively. Patients were treated mostly with monotherapy (37.7%) or 2-drug anti-hypertensive combination (38.7%). Factors associated with prevalence of hypertension included age; smoking; body mass index; physical exercise; family history of hypertension; hyperuricaemia; and CKD. Control rate was associated with CKD stage, BP monitoring at home, and use of drug combinations. Despite high rates of awareness and treatment, the control rates are low. CKD stages 4 and 5 adversely affect the control rate. The results suggest the immediate need of comprehensive controlling measures to improve the control of hypertension in Chinese patients with CKD.

Hypertension and chronic kidney disease (CKD) are interrelated and increasingly recognised as a serious global public health concern^1^. The estimated worldwide prevalence of CKD is 8% to16%^2^. Its prevalence is not only high in developed countries^3^,^4^,^5^ but is also on the rise in developing countries^6^,^7^. In China, an estimated 10.8% of the population has CKD, and the prevalence has been observed to increase with increasing age^7^,^8^.

Hypertension is also highly prevalent in China (≈26.6%) and predominantly in men^9^. Hypertension became one of the most prominent risk factors and a comorbidity of CKD over a period of time (from 1990s to 2009–2010), and 24.2% elderly patients develop CKD as a result of hypertension^10^. In China, 2 studies reported the prevalence of hypertension in patients with CKD at 82.0% and 67.3%, respectively. However, despite high awareness and treatment rates reported in these studies, the control rates for hypertension were relatively very low^11^,^12^. Moreover, the control rate of hypertension is inversely associated with the CKD stage^13^. Uncontrolled hypertension is associated with poor prognosis of CKD, its progression to end-stage renal disease (ESRD), renal failure, and mortality. Patients with high-grade hypertension are at a higher risk of CKD progression^14^ and other complications such as stroke, cardiovascular disease, and target organ damage (TOD)^15^. Although awareness and treatment of hypertension in patients with CKD are showing an improving trend, treatment and control of blood pressure (BP) at all the CKD stages remain suboptimal^16^.

Despite the recognition of hypertension and CKD as a major public health concern, epidemiologic research to provide insights into public health approaches on prevention and treatment of hypertension in patients with CKD is limited and very few studies have been conducted in China to determine the prevalence and control rate of hypertension in patients with CKD. Therefore, we determined the prevalence, awareness, control, and rate of reaching the target BP in hypertensive Chinese patients with CKD using a nationwide survey across China. We study also evaluated the anti-hypertensive drug use situation, pattern of expenditure on drugs and factors associated with hypertension prevalence and control.

## Results

### Patient Characteristics

The study enrolled 6079 patients with CKD (mean age, 51.0 ± 16.37 years; mean BMI, 23.49 ± 3.483 kg/m^2^), with more than 50% (51.9%) males. The study population had 27.1% patients with CKD stage 5. The main aetiologies of CKD included diabetic nephropathy (n = 695, 11.4%); hypertensive and ischaemic renal damage (n = 875, 14.4%); primary chronic glomerulonephritis (n = 3710, 61.0%); secondary glomerulonephritis (n = 403, 6.6%); interstitial disease of renal tubules (n = 116, 1.9%); cystic kidney disease (n = 94, 1.5%); obstructive nephropathy (n = 56, 0.9%); hereditary and congenital nephropathy (n = 32, 0.5%); and tumour-related renal damage (n = 9, 0.1%). CKD was of unknown origin in 209 patients (3.4%). Of the total participants, 4328 were found to be hypertensive (71.2%). Other demographic characteristics and medical history of the patients are presented in Table 1. Mean BP at baseline was 136.6/81.3 mmHg (±19.55/11.27) for the whole study population (n = 6078). Other than hypertension, the major co-morbid indications were dyslipidaemia (34.6%), hyperuricaemia (32.6%), diabetes (21.6%), coronary heart disease (6.8%), other endocrine disorders (18.6%), and stroke (6%).

### Prevalence, Awareness, Treatment, and Control Rates of Hypertension

**Figure 1:**
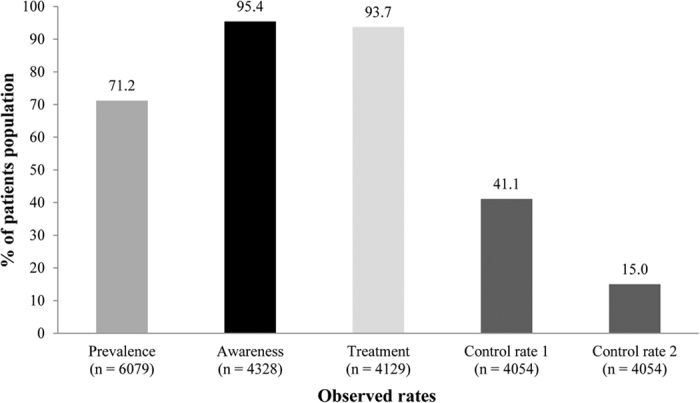
Prevalence, Awareness, and Control Rates of Hypertension.

**Figure 2:**
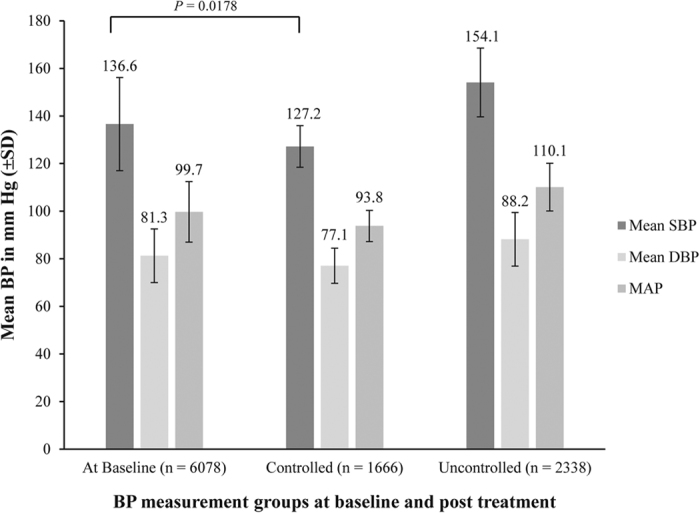
SBP, DBP, and Mean Arterial Pressure at Baseline and Post Treatment.

Overall, 4328 of the 6079 (71.2%) patients had hypertension. Prevalence of grade 1, 2, 3, and isolated systolic hypertension were 27.8%, 11.3%, 2.9%, and 22.9%. The overall prevalence, awareness, treatment and control rates are presented in Fig. 1. A total of 93 (2.3%) patients were diagnosed with hypertension after use of cortical hormone (n = 55, 1.3%), Chinese medicines (n = 17 0.4%), or other medicines (n = 25, 0.6%). Hypertension in these patients was possibly due to administration of previously administered therapy. Low prevalence of grade 3 in patients with CKD and high prevalence of patients with CKD stage 5 may indicate that small number of patients with CKD stage 5 have grade 3 hypertension. Among patients with hypertension, 4129 (95.4%) had been diagnosed and were aware of the disease before the study. The rate of treatment was 93.7%. Control rate 1 and control rate 2 were achieved by 41.1% and 15.0% patients, respectively with anti-hypertensive medications. Figure 2 shows reduction of BP post treatment from baseline. Among patients who were taking anti-hypertensive medications, BP was controlled in 1666 patients and lowered to a mean of 127.2/77.1 mmHg (±8.74/7.36). The decrease in BP in the controlled group was significant compared with baseline (difference from baseline: −9.4/4.2 mmHg, *P* = .0178). A total of 1778 patients had uncontrolled hypertension (did not achieve control rate 1 or 2). In these patients, BP at end of study was higher compared with the baseline mean BP, 154.1/88.2 mmHg (±14.44/11.28), difference: +17.5/6.9 mmHg]).

Patients who were aware of the disease also used non-drug treatments such as restricting salt intake (73.7%), controlling body weight (58.7%), cessation of smoking (17.7%) and alcohol consumption (24.3%), and starting physical activity (31.4%). Most of the patients (97.0%) started with anti-hypertensive therapy after diagnosis, and majority of the patients (85.3%) took the medications regularly.

### Pattern of Anti-hypertensive Administration and Drug Expenditure

**Figure 3:**
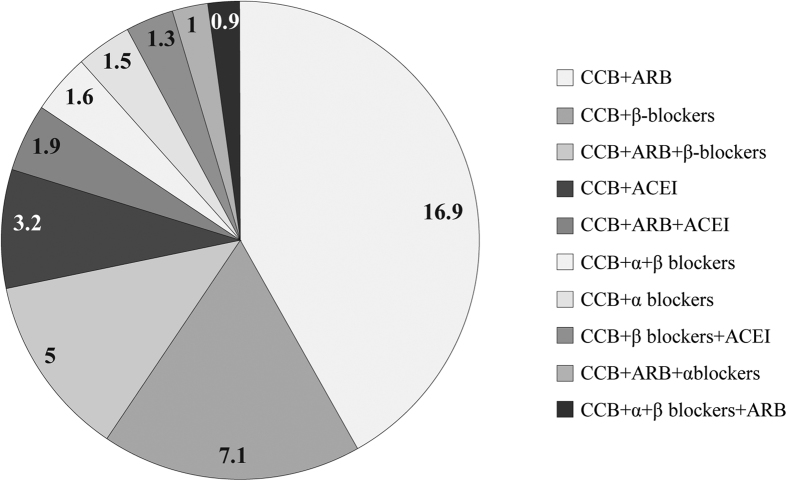
Percentage Usage of CCBs in Combination Therapy.

**Figure 4:**
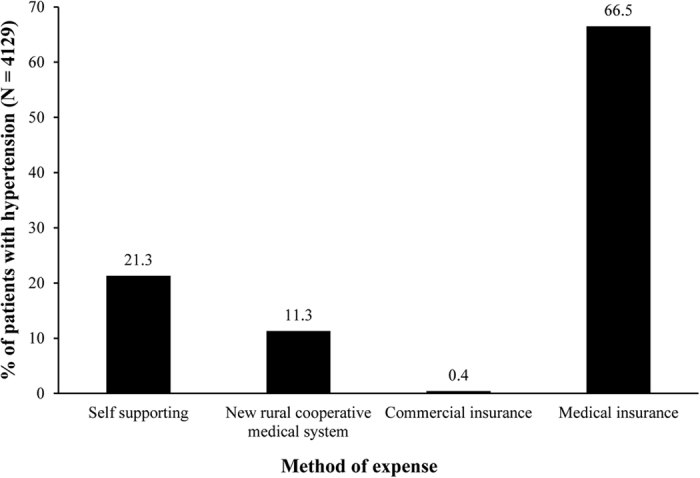
Anti-Hypertensive Drug Expenditure Patterns.

Monotherapy (37.7%) and 2 drug combination (38.7%) were the preferred anti-hypertensive treatment regimen. Three-, 4-, 5-, and 6-drug combinations were received by 15.8%, 3.9%, 0.7%, and 0.1% patients, respectively. Patients were administered angiotensin receptor blockers (ARB), α- and β-adrenergic blockers, calcium channel blocker (CCB), diuretics, angiotensin converting enzyme inhibitors (ACEI), centrally acting medicines, or compound preparations. Overall, CCB was the most preferred drug class in China (77.6%), followed by drugs affecting renin angiotensin system inhibitors (RASI; ARB [52.9%] and ACEI [24.0%] and β-receptor inhibitors [24.0%]). Combination therapy was administered in 2406 patients. CCBs were the major component of the drug combinations. A summary of CCB use in combinations is presented in Fig. 3. Anti-hypertensive drug expenditure patterns of patients are presented in Fig. 4. Patients spending on drugs through public medical insurance had employment (20.8%), retirement (38.8%), or small town insurance (6.9%).

### Factors Associated With Prevalence, Awareness, Treatment, and Control Rates

All the independent factors were evaluated for association with prevalence, awareness, treatment, and control rates of hypertension using logistic regression. The analysis revealed that age, region or geography, smoking, BMI, lack of physical exercise, history of hypertension, hyperuricaemia; and stages of CKD had a significant positive association with prevalence of hypertension. In addition, awareness and treatment rates of hypertension were associated with age, region/geography, and glomerular filtration rate (GFR) staging. Apparently, level of education did not show any association with disease awareness and treatment. Control rates 1 and 2 for hypertension had association with age, gender, region, smoking, BMI, GFR staging, and self-monitoring of BP at home.

Multivariate analysis of the associated factors (Table 2) confirmed that patients 45 to 65 and 65 to 80 years of age had a significantly higher prevalence compared with patients 18 to 45 years of age (*P* < 0.001, for both comparisons). Prevalence of hypertension was higher in middle and northern China compared with southern China (*P* < 0.0001, for both comparisons). The prevalence of hypertension was significantly associated with 18–23 kg/m^2^, 23–28 kg/m^2^ and 28–32 kg/m^2^ higher BMI groups compared with the <18 kg/m^2^ BMI group (*P* = 0.0059, <0.0001, and <0.0001, respectively), with higher BMI showing greater association with hypertension prevalence. Compared with stage 1 CKD (reference stage), all the other stages of CKD had higher probability of being hypertensive (stage 2 OR: 1.387, *P* = 0.0004; stage 3a OR: 2.608, *P* < 0.0001; stage 3b OR: 2.705, *P* < 0.0001; stage 4 OR: 6.139, *P* < 0.0001; and stage 5 OR: 10.205, *P* < 0.0001). Lack of physical exercise, family history of hypertension and co-morbid indications increased the likelihood of hypertension in patients with CKD. Older age groups had a significant greater awareness of disease than the 18- to 45-year group (*P* < 0.001). Awareness rate was significantly associated with family history of hypertension (odds ratio (OR), 2.576; confidence interval [CI], 1.779–3.731; *P* < 0.0001). All the age groups and regions had a significant association with rate of treatment. Control rate 1 was significantly dependent on the region (for mid China vs. south China: OR, 3.028; CI, 2.207–4.153; *P* < .0001; for north China vs. south China: OR, 1.609; CI, 1.121–2.309; *P *= 0.0099) and smoking (OR, 0.754; CI, 0.522–1.778; *P *=* *0.0077). Likelihood of achieving control rate 1 (≤140/90 mmHg) was significantly lower in patients with stage 5 CKD (OR, 0.559; CI, 0.404–0.774; *P *=* *0.0005) and stage 4 CKD (OR, 0.605; CI, 0.404–0.905; *P *=* *0.0145) compared with stage 1 CKD, which interpreted that the patients with uncontrolled hypertension belonged to stage 4 and 5 CKD. In stages 2, 3a and 3b, probability of achieving control rate 1 was similar to the CKD stage 1. Patients who applied home BP measurement (OR, 1.649; CI, 1.3–2.091; *P* < 0.0001) and used drug combination (OR, 0.597; CI, 0.483–0.74; *P* < 0.0001) were more likely to achieve a BP of <140/90 mmHg. Achievement of control rate 2 was dependent on gender (OR, 1.466; CI, 1.098–1.957; *P *=* *0.0095), BP monitoring at home (OR, 1.879; CI, 1.333–2.648; *P *=* *0.0003), and use of drug combination (OR, 0.548; CI, 0.415–0.722; *P* < 0.0001).

## Discussion

The prevalence of hypertension and CKD is constantly increasing in China and has seen multiple fold increase over the past 3 decades^17^,^18^,^19^. Since there are only a few studies that have determined the epidemiology and control of hypertension in Chinese patients with CKD; reliable information is necessary for the development of health policies in China, to prevent and control hypertension in patients with CKD. As per our knowledge, this is one of the largest nationwide survey that analysed the prevalence of hypertension, awareness of the disease, rates of treatment, control of hypertension; anti-hypertensive drug use and drug expenditure pattern; and factors associated with prevalence, awareness, treatment, and control rates of hypertension in patients with CKD in China.

Nationwide surveys such as the Chronic Renal Insufficiency Cohort (CRIC) study, Chronic REnal Disease In Turkey (CREDIT) study, and study in Columbia revealed that the prevalence, awareness, and treatment rates for hypertension were high and the control rates were sub-optimal^20^,^21^,^22^. Data from the CRIC study showed prevalence of 85.7%, awareness rate of 98.9%, treatment rate of 98.3% and control rate of 67.1% (<140/90 mmHg) and 46.1% < 130/80 130/80 mmHg^20^. The CREDIT study also reported a prevalence rate of 56.3%, awareness and treatment rates of 56.3%, 61.9% and 44.2%. The control rate was sub-optimal at 28.8% after treatment^21^. Sarafidis *et al*. reported a control rate of 13.2% despite high prevalence (86.2%), awareness (80.2%) and treatment rates (70.0%)^22^. Studies determining the control of hypertension in the United Kingdom and Japan also reported high prevalence (88.0% and 58.0%) and low control rates of hypertension (34.2% and 34.6%) in patients with CKD^23^,^24^,^25^. Similarly, a multinational survey was conducted in 2009 to 2010 across China that reported hypertension prevalence, awareness, and treatment rates of 82.0%, 90.7%, and 87.3%, respectively, in adult Chinese patients with CKD. The control rates were however very low at 29.6% for target BP of <140/90 mmHg and 12.1% for target BP of <130/80 mmHg. Another study by Zheng *et al*. showed that the rates of prevalence of hypertension in patients with CKD, awareness, and treatment were 67.3%, 85.8%, and 81.0%, respectively. The control rates of hypertension were 33.1% for BP <140/90 mmHg and 14.1% for BP <130/80 mmHg^12^. On similar lines, our study also reported high rates of prevalence of hypertension, awareness, and treatment. The control rate for target BP of <140/90 mmHg was observed to be slightly higher in this study than the previous studies (41.1% vs 29.6% and 33.1%). Proportion of patients achieving target BP of <130/80 mmHg was similar in our study and earlier studies (15.0% vs 12.1% and 14%). Low control rates in all the studies showed that hypertension in China is not optimally controlled in patients with CKD. This is an alarming situation considering the continuously increasing trend of hypertension in Chinese patients with CKD. The study also highlights relatively very low control rates of hypertension despite increased awareness and treatment. The control rate among different countries might vary due to ethnicity, economic or educational level differences, as reported by Sarafidis *et al*. Within the same country, improvement in economy and setting of the study (study conducted in rural or urban setting) may also improve the treatment and control rates.

The Chinese guidelines for the treatment of hypertension suggest the use of CCB, ACEI, ARB, diuretics, and β-blockers as monotherapy or combination therapy for hypertension management^15^. The JNC8 guidelines recommend the use of ACEI or ARB for CKD patients with hypertension^26^, whereas the European Society of Hypertension (ESH) guidelines recommend the use of all anti-hypertensive drugs except diuretics (in the haemodialysis patients) after dose determination based on hemodynamic instability and the ability of the drug to be dialysed^27^. In the present study, CCB, ARB, and β-blockers were the most prescribed medications in China.

Anti-hypertensive drug expenditure patterns also support the association of prevalence, awareness, and treatment of hypertension with older age, as approximately 39% patients were retired from work.

The factors associated with prevalence, control, awareness, and treatments were determined. The risk of hypertension was more in older patients (42–65 and 65–80 years of age) than in relatively younger patients (18–45 years of age). Hypertension prevalence was associated more with patients from middle and southern China than those from north China. Smokers, patients with BMI >18 kg/m^2^, lack of physical exercise, CV and metabolic co-morbidities, and CKD stages 2 to 5 were associated with a high prevalence of hypertension. In a study by Stevens *et al*., the prevalence of hypertension in participants with CKD aged 65 and older in KEEP and NHANES 1999 to 2006 were 94.5% and 91.6%, respectively^28^. Findings from this study were consistent with previous literature that showed older age, higher BMI, smoking, CV, and metabolic disease as the factors associated with a high prevalence of hypertension^11^,^22^. Awareness of hypertension was found to be higher in older patients and those with a family history of hypertension. Older age and geography were associated with a higher rate of treatment. Control rate 1 was determined primarily by CKD stage, BP monitoring at home, and use of drug combinations for treatment. Patients with CKD stage 4 and stage 5 were not able to achieve control rate 1. Anti-hypertensive therapy with ARBs as mono- or combination therapy improved the rate of hypertension control in Chinese patients with hypertension and CKD. Measurement of BP at home was responsible for increasing the awareness of patients toward the disease and medication, subsequently improving the BP control rate. Achievement of control rate 2 was positively associated with female gender, regular BP monitoring at home, and use of combination drug therapy. Overall, the control rate was low for patients taking anti-hypertensive medications. The findings highlight that high prevalence and control of hypertension were associated with renal function in adult patients with CKD.

The present study has the following strengths: (i) stringent use of a standard data collection protocol; (ii) inclusion of extensive data on demography, medical-related history, drug treatments, expenditure behaviour of patients, and factors associated with hypertension in patients with CKD; and (iii) analyses of data from a large sample size.

This study has certain limitations because of which the findings must be interpreted with caution. Most notably, the cross-sectional study design does not allow for causal associations to be established with certainty. Second, sampling was not done randomly. Number of patients was limited and out of those, ESRD patients accounted for a relatively large number. Third, potential limitation is that there might be some variation in evaluating laboratory parameters or BP measurement despite following the standardised protocol. Selection of hospitals from 3 developed cities of China limited the extrapolation of the results to the whole Chinese CKD population. Another potential limitation of our study is that we correlated the control of hypertension with GFR only, while the correlation between hypertension and albuminuria was not evaluated. Furthermore, well-designed, large sample cohort studies and randomised clinical trials are warranted to draw effective conclusions.

The prevalence, awareness, and treatment rates of hypertension in Chinese patients with CKD were high. However, the low control rates reflect the sub-optimal control of hypertension in patients with CKD. Association of monitoring BP indicated toward the need of greater awareness of the disease, whereas association of drug combinations with control rates suggested toward the need of developing effective interventions for managing hypertension in patients with CKD in China. Therefore, strategies to improve awareness and treatment of hypertension in patients with CKD are needed in China for better healthcare management.

## Methods

### Study Design and Population

The ‘Research on hypertensive nephropathy and ischemic kidney diseases National key technology R&D program (12-5) (Study No. 2011BAI10B00 (2011BAI10B06)’ study was one of the largest multicentre, cross-sectional survey in China. A total of 22 hospitals (Supplementary Table S1) were selected from mid-China (n = 11), northern (n = 6), and southern China (n = 5).

Patients with CKD (aged >15 years) were invited to participate in this survey. The inclusion criteria were: (i) age 16 to 85 years; (ii) CKD (stage 1–5) with or without hypertension. Patients were excluded if they had malignant tumour, BMI >32 kg/m^2^; coagulation disorder, drug abuse history or addicted to alcohol, severe CV or cerebrovascular disease, psychological disorder or were pregnant or lactating. The study was approved by the ethics committee (Ref no: 2012–54) of all the 22 hospitals in China (Rui Jin Hospital Shanghai Jiao Tong University School of Medicine, Shanghai; Huashan Hospital Fudan University, Shanghai; Shanghai General Hospital, Shanghai; Jiangsu Province Hospital, Jiangsu; Sichuan Provincial People’s Hospital, Sichuan; Union Hospital Tongji Medical College, Hubei; Tongji Hospital Tongji Medical College, Hubei; Jiangsu Taizhou People’s Hospital, Jiangsu; Yangpu Hospital, Shanghai; Shanghai Construction Group Hospital, Shanghai; Tongren Hospital Shanghai Jiao Tong University School Of Medicine, Shanghai; Guangdong General Hospital, Guangdong; Fujian Provincial Hospital, Fujian; The First Affiliated Hospital of Nanchang University, Jiangxi; The First Affiliated Hospital of Wenzhou Medical University, Zhejiang; The First Affiliated Hospital of Xiamen University, Fujian; Beijing Anzhen Hospital Capital Medical University, Beijing; PLA Navy General Hospital, Beijing; The First Affiliated Hospital of Zhengzhou University, Henan; The First Affiliated Hospital of Xinjiang Medical University, Xinjiang; Inner Mongolia People’s Hospital, Neimenggu; The 2^nd^ Affiliated Hospital of Harbin Medical University, Heilongjiang) and conducted in accordance with International Conference on Harmonisation Good Clinical Practice (GCP, E6), Declaration of Helsinki (1964) and its subsequent revisions. All participants provided their informed consent before initiation of the study.

### Data Collection

**Figure 5:**
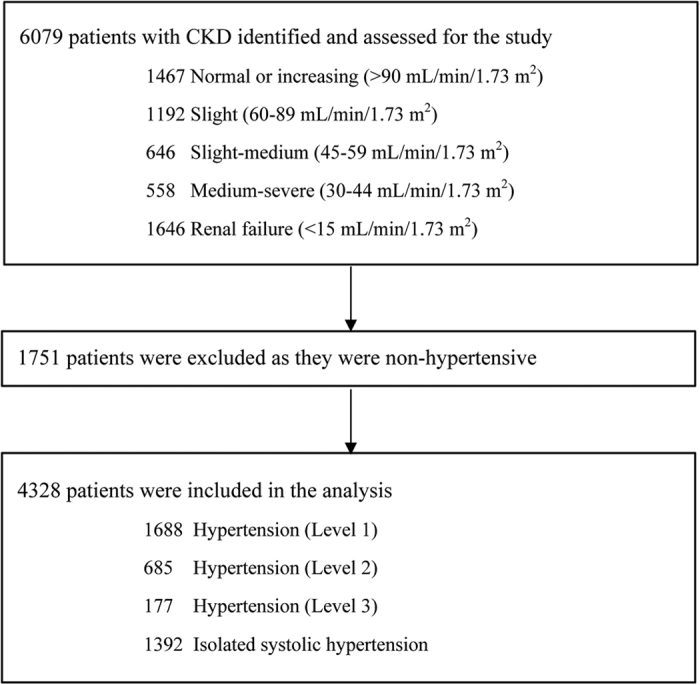
Study Flow Chart.

The data were collected from 30 June 2012 to 30 December 2013 (18 months) using a standardised questionnaire. Before commencing the study, all investigators were trained on the study protocol. Trained investigators collected the demographic, clinical, laboratory, drug treatment (use of monotherapy or combination), drug expense, and BP data. In addition, the investigators completed case report form using a standardised protocol. The study flow diagram is presented in Fig. 5.

BP was measured by trained healthcare professionals (HCPs) in the morning (8 am to 11 am) as per standard protocol. Literature suggests taking a minimum 2 measurements for ensuring the accuracy of BP measurement. Therefore, before recording the BP, participants rested for approximately 5 minutes following which BP was measured twice using a mercury sphygmomanometer with at least a 1-minute interval between measurements in an ambulatory setting. Mean of the 2 measurements was considered for the analysis. If the difference between the 2 BP measurements was >5 mmHg, one more measurement was taken, and mean of the 3 BP measurements was used^29^,^30^.

### Definitions of Hypertension, Awareness, Treatment, and Control Rates

Hypertension is defined as a systolic BP (SBP) of ≥140 mmHg or a diastolic BP (DBP) of ≥90 mmHg or undergoing treatment for hypertension. Grades 1, 2, and 3 of hypertension are defined as BP of 140–159/90–99, 160–179/100–109, and ≥180/≥110 mmHg. Participants with SBP of 130 to 139 mmHg or DBP of 85 to 89 mmHg were considered to be having critical or borderline hypertension, as it is not suitable to define these patients as normotensives. Isolated systolic hypertension was defined as BP ≥140/<90 mmHg^27^. The ESH and the European Society of Cardiology (ESC) suggest maintaining a BP of <140/90 mmHg in patients with CKD, whereas the Chinese 2010 Hypertension guidelines suggest maintaining BP of <130/80 mmHg in such patients^15^,^27^. It is not possible to recommend specific BP targets in elderly adults with CKD due to lack of evidence and difference in target BP range of various guidelines^11^. Therefore, in this study target BP levels were chosen as <140/90 (control rate 1; as per ESH/ESC, Joint National Committee on Prevention, Detection, Evaluation, and Treatment of High BP [JNC-8] Guidelines)^26^,^27^ and <130/80 mmHg (control rate 2; Chinese 2010 Hypertension guidelines)^15^ with anti-hypertensive drug treatment. Awareness rate was defined as the proportion of patients with hypertension who were diagnosed with hypertension by a physician or HCP before enrolment into this study. Treatment rate was defined as the proportion of patients with hypertension receiving anti-hypertensive medications before enrolment in the study.

### Definition of CKD

In accordance with the Kidney Disease Outcomes Quality Initiative (KDOQI) guidelines, CKD was defined as GFR of <60 mL/min/1.73 m^2^ for ≥3 months. Patients were classified into different CKD stages based on their GFR. Definition of different stages of CKD is presented in Table 1^31^.

### Statistical Analysis

Statistical analysis was performed using Statistical Analysis System (SAS) Ver. 9.4 (SAS Institute Inc., Cary, NC, USA). Descriptive statistics was used to evaluate demographic characteristics, medical history, and parameters for laboratory inspection, drug treatment, and expenditure patterns. Logistic regression was used to find the association between demographic and clinical factors with prevalence, awareness, and control rates. A *P* value of <0.05 was considered as statistically significant.

## Additional Information

**How to cite this article**: Zhang, W. *et al*. A nationwide cross-sectional survey on prevalence, management and pharmacoepidemiology patterns on hypertension in Chinese patients with chronic kidney disease. *Sci. Rep.* **6**, 38768; doi: 10.1038/srep38768 (2016).

**Publisher's note:** Springer Nature remains neutral with regard to jurisdictional claims in published maps and institutional affiliations.

## Supplementary Material

Supplementary Table S1

## Figures and Tables

**Table 1:** Demographic Characteristics.

Demographic Characteristics	Value, n (%)
Total number of patients	6079 (100)
Age, years (±SD)	51.0 (±16.37)
Men	3154 (51.9)
Ethnic group/nationality	
Han	5964 (98.1)
Others	115 (1.9)
Educational level	
Primary school and below	1218 (20.0)
Junior middle school	1671 (27.5)
Senior high school	1227 (20.2)
Technical secondary school	440 (7.2)
Junior college	635 (10.4)
Undergraduate college or above	879 (14.5)
Smoking history	
No	4719 (77.6)
Yes	1360 (22.4)
Has quit smoking	724 (11.9)
Still smoking	635 (10.4)
Physical exercise	
No	4508 (74.2)
Yes	1571 (25.8)
Mean serum creatinine (mg/dL)	3.5 (4.15)
Mean urine protein positive	4606 (75.8)
Mean urine ACR (mg/mmol) (n = 4154)	165.6 (382.65)
Proteinuria level	
A1 (<30 mg/24 h)	501 (8.2)
A2 (30–300 mg/24 h)	1475 (24.3)
A3 (>300 mg/24 h)	2475 (40.7)
Mean eGFR (MDRD), mL/min	54.8 (43.64)
Mean eGFR (CKD-EPI), mL/min	53.1 (40.62)
**GFR staging (mL/min/1.73 m**^**2**^)	
Stage 1: Normal or increasing (>90)	1467 (24.1)
Stage 2: Slight (60–89)	1192 (19.6)
Stage 3a: Slight-medium (45–59)	646 (10.6)
Stage 3b: Medium-severe (30–44)	558 (9.2)
Stage 4: Severe (15–29)	569 (9.4)
Stage 5: Renal failure (<15)	1646 (27.1)
**Albuminuria (mg/g)**	
<30	941 (15.5)
30–300	1736 (28.6)
>300	2636 (43.4)

ACR = albumin and albumin/creatinine ratio, CKD-EPI = chronic kidney disease - epidemiology collaboration, eGFR = estimated glomerular filtration rate, MDRD = modification of diet in renal disease.

**Table 2:** Result of Multi-factor Regression Analysis.

Characteristic	Prevalence		Awareness		Treatment		Control Rate 1		Control Rate 2	
	OR (95% CI)	*P* Value	OR (95% CI)	*P* Value	OR (95% CI)	*P* Value	OR (95% CI)	*P* Value	OR (95% CI)	*P* Value
Age ([18, 45] -[65, 80])	3.659 (2.96–4.522)	<0.0001	2.381 (1.573–3.604)	<0.0001	3.393 (1.962–5.869)	<0.0001	—	—	—	—
Age ([18, 45]-[45, 65])	1.868 (1.606–2.172)	<0.0001	1.613 (1.162–2.239)	0.0042	2.27 (1.498–3.442)	<0.0001	—	—	—	—
Gender, male-female	—	—	—	—	—	—	—	—	1.466 (1.098–1.957)	0.0095
Region: South-middle	1.598 (1.359–1.879)	<0.0001	0.878 (0.588–1.31)	0.5229	0.54 (0.31–0.941)	0.0295	3.028 (2.207–4.153)	<0.0001	3.69 (2.273–5.99)	<0.0001
Region: South-north	1.982 (1.601–2.454)	<0.0001	0.316 (0.213–0.468)	<0.0001	0.259 (0.146–0.46)	<0.0001	1.609 (1.121–2.309)	0.0099	0.963 (0.522–1.778)	0.9053
Smoke (no-yes)	1.499 (1.253–1.794)	<0.0001	—	—	—	—	0.754 (0.613–0.928)	0.0077		
BMI ([0, 18]-[28, 32])	3.707 (2.557–5.374)	<0.0001	—	—	—	—	—	—	—	—
BMI ([0, 18]-[23, 28])	2.415 (1.786–3.267)	<0.0001	—	—	-	—	—	—	—	—
BMI ([0, 18]-[18, 23])	1.513 (1.126–2.032)	0.0059	—	—	—	—	—	—	—	—
Patients have physical exercise or not (no-yes)	1.522 (1.292–1.793)	<0.0001	—	—	—	—	—	—	—	—
Have family history of hypertension or not (no-yes)	2.594 (2.201–3.057)	<0.0001	2.576 (1.779–3.731)	<0.0001			—	—	—	—
Have other endocrine diseases or not (no-yes)	1.248 (1.029–1.515)	0.0247	—	—	—	—	—	—	—	—
Have coronary heart disease or not (no-yes)	1.994 (1.26–3.157)	0.0032	—	—	—	—	—	—	—	—
Have heart failure or not (no-yes)	2.912 (1.387–6.113)	0.0047	—	—	—	—	—	—	—	—
Have hyperuricaemia or not (no-yes)	1.566 (1.322–1.854)	<0.0001	—	—	—	—	—	—	—	—
Have stroke or not (no-yes)	4.215 (2.348–7.567)	<0.0001	—	—	—	—	—	—	—	—
GFR staging 1: normal or increased (>90) −5: renal failure (<15)	10.205 (8.2–12.701)	<0.0001	—	—	—	—	0.559 (0.404–0.774)	0.0005	—	—
GFR staging 1: normal or increased (>90) −4: severe (15–29)	6.139 (4.546–8.291)	<0.0001	—	—	—	—	0.605 (0.404–0.905)	0.0145	—	—
GFR staging 1: normal or increased (>90) −3b: middle-severe (30–44)	2.705 (2.075–3.526)	<0.0001	—	—	—	—	0.957 (0.631–1.452)	0.8358	—	—
GFR staging 1: normal or increased (>90) −3a: mild-middle (45–59)	2.608 (2.054–3.311)	<0.0001	—	—	—	—	0.903 (0.611–1.335)	0.6089	—	—
GFR staging 1: normal or increased (>90) −2: mild (60–89)	1.387 (1.157–1.662)	0.0004	—	—	—	—	1.05 (0.735–1.501)	0.7875	—	—
Test BP in house or not	—	—	—	—	—	—	1.649 (1.3–2.091)	<0.0001	1.879 (1.333–2.648)	.0003
Drug combination administered (no-yes)	—	—	—	—	—	—	0.597 (0.483–0.74)	<0.0001	0.548 (0.415–0.722)	<0.0001

BMI = body mass index, GFR = Glomerular filtration rate, OR = odds ratio.
