# Supplementary material for: A nationwide cross-sectional survey on prevalence, management and pharmacoepidemiology patterns on hypertension in Chinese patients with chronic kidney disease
Source: Sci Rep. 2016 Dec 20;6:38768. doi: 10.1038/srep38768 (PMC5171924; doi:10.1038/srep38768)
Supplement: Supplementary Table S1 [file srep38768-s1.doc]

**A nationwide cross-sectional survey on prevalence, management and pharmacoepidemiology patterns on hypertension in Chinese patients with chronic kidney disease**

Wen Zhang, MD^1^; Wei Shi, MD^2^; Zhangsuo Liu, MD^3^; Yong Gu, MD^4^; Qinkai Chen, MD^5^; Weijie Yuan, MD^6^; Yanlin Zhang, MD^7^; Li Gong, MD^8^; Rong Zhou, MD^9^; Mingxu Li, MD^10^; Hong Cheng, MD^11^; Jian Liu, MD^12^; Jun Cen, MD^13^; Chaoxing Huang, MD^14^; Yeping Ren, MD^15^; Peiju Mao, MD^16^; Changying Xing, MD^17^; Fuyuan Hong, MD^18^; Dongsheng Jiang, MD^19^; Li Wang, MD^20^; Gang Xu, MD^21^; Jianshe Liu, MD^22^; and Nan Chen, MD^1*^

^1^Department of Nephrology, Ruijin Hospital Shanghai Jiao Tong University School of Medicine, Shanghai, 200025, China

^2^Department of Nephrology, Guangdong General Hospital, Guangzhou, 510030, China

^3^Department of Nephrology, The First Affiliated Hospital of Zhengzhou University, Zhengzhou, 450052 China

^4^Department of Nephrology, The Fifth Affiliated Hospital of Fudan University, Shanghai, 200240, China ^5^Department of Nephrology, The First Affiliated Hospital of Nanchang University, Nanchang, 330006, China

^6^Department of Nephrology, The First Affiliated Hospital of Shanghai Jiao Tong University, Shanghai, 200080, China

^7^Department of Nephrology, The First Affiliated Hospital of Xiamen University, Xiamen, 361003, China

^8^Department of Nephrology, Department of Nephrology, Inner Mongolia People’s Hospital, Inner Mongolia, China

^9^Department of Nephrology, Shanghai Yangpu Hospital, Tongji University, Shanghai, 200090, China

^10^Department of Nephrology, PLA Navy General Hospital, Beijing, 100048, China

^11^Department of Nephrology, Beijing Anzhen Hospital Capital Medical University, Beijing, 100029, China

^12^Department of Nephrology, The First Affiliated Hospital of Xinjiang Medical University, Xinjiang, China

^13^Department of Nephrology, Shanghai Construction Group Hospital, Shanghai, 200083, China

^14^Department of Nephrology, The First Affiliated Hospital of Wenzhou Medical University, Wenzhou, 325000, China

^15^Department of Nephrology, The Second Affiliated Hospital of Harbin Medical University, Harbin, China

^16^Department of Nephrology, Tongren Hospital, Shanghai Jiao Tong University School of Medicine, Shanghai, 200336, China

^17^Department of Nephrology, Jiangsu Province Hospital, Nanjing, 210029, China

^18^Department of Nephrology, Fujian Provincial Hospital, Fuzhou, 350001, China

^19^Department of Nephrology, Jiangsu Taizhou People’s Hospital, Taizhou, 225300, China

^20^Department of Nephrology, Sichuan Provincial People’s Hospital, Sichuan, 610072, China

^21^Department of Nephrology, Tongji Hospital, Tongji Medical College, Huazhong University of Science and Technology, Wuhan, 430030, China

^22^Department of Nephrology, Union Hospital, Huazhong University of Science and Technology, Wuhan, 430022, China

***Correspondence To:**

**Dr. Nan Chen**

Department of Nephrology,

Ruijin Hospital, Shanghai Jiao Tong University School of Medicine.

197 Ruijin Er Road, Huangpu District, Shanghai, 200025, China

Tel: +86 13601638963; Fax number: +86 2164456419

E-mail: [chen-nan@medmail.com.cn](mailto:chen-nan@medmail.com.cn)

**Supplementary Tables**

Table S1 Study Sites

| **Region** | **Name of center** | **Province** | **Patients enrolled (cases)** |
| --- | --- | --- | --- |
| Mid China | Rui Jin Hospital Shanghai Jiao Tong University School of Medicine | Shanghai | 1599 |
| Mid China | Huashan Hospital Fudan University | Shanghai | 430 |
| Mid China | Shanghai General Hospital | Shanghai | 343 |
| Mid China | Jiangsu Province Hospital | Jiangsu | 80 |
| Mid China | Sichuan Provincial People's Hospital | Sichuan | 60 |
| Mid China | Union Hospital Tongji Medical College Huazhong University of Science and Technology | Hubei | 41 |
| Mid China | Tongji Hospital Tongji Medical College Huazhong University of Science and Technology | Hubei | 50 |
| Mid China | Jiangsu Taizhou People's Hospital | Jiangsu | 64 |
| Mid China | Yangpu Hospital, Tongji University | Shanghai | 200 |
| Mid China | Shanghai Construction Group Hospital | Shanghai | 102 |
| Mid China | Tongren Hospital Shanghai Jiao Tong University School Of Medicine | Shanghai | 100 |
| South China | Guangdong General Hospital | Guangdong | 1000 |
| South China | Fujian Provincial Hospital | Fujian | 80 |
| South China | The First Affiliated Hospital of Nanchang University | Jiangxi | 392 |
| South China | The First Affiliated Hospital of Wenzhou Medical University | Zhejiang | 100 |
| South China | The First Affiliated Hospital of Xiamen University | Fujian | 300 |
| North China | Beijing Anzhen Hospital Capital Medical University | Beijing | 6 |
| North China | PLA Navy General Hospital | Beijing | 179 |
| North China | The First Affiliated Hospital of Zhengzhou University | Henan | 506 |
| North China | The First Affiliated Hospital of Xinjiang Medical University | Xinjiang | 138 |
| North China | Inner Mongolia People's Hospital | Neimenggu | 209 |
| North China | The 2nd Affiliated Hospital of Harbin Medical University | Heilongjiang | 100 |
